# Spontaneous Hemoperitoneum from Rupture of Massive Leiomyoma

**DOI:** 10.5811/cpcem.2017.1.33190

**Published:** 2017-03-16

**Authors:** Arielle Schwitkis, Steven Shen, Elaine Vos, Sam S. Torbati

**Affiliations:** * Cedars-Sinai Medical Center, Department of Emergency Medicine, Los Angeles, California; † Western Michigan University Homer Stryker M.D. School of Medicine, Kalamazoo, Michigan

## CASE REPORT

**Image 1:**
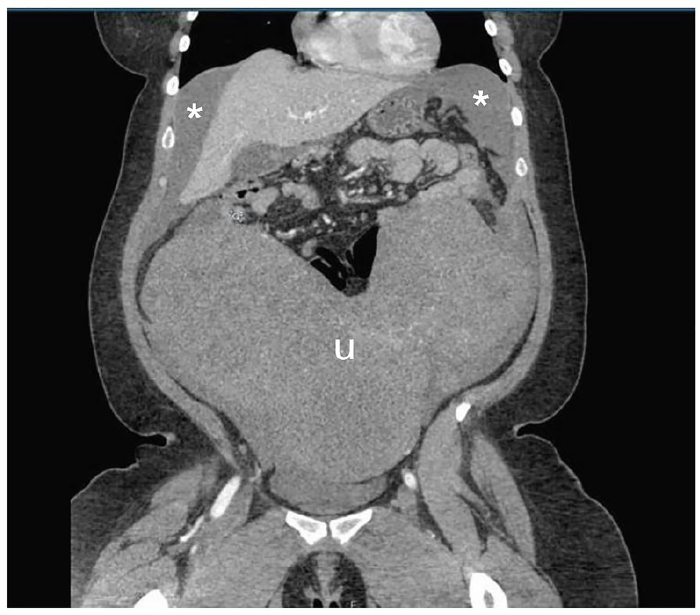
Abdominal computed tomography, coronal view, demonstrating massive fibroid filled uterus (u) and hemoperitoneum (asterisks).

**Image 2:**
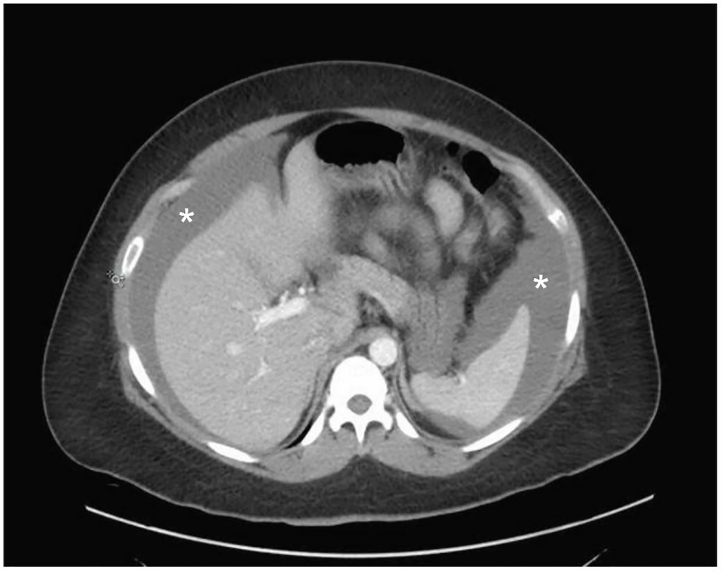
Abdominal computed tomography, cross section, demonstrating location of hemoperitoneum (asterisks).

A 34-year-old woman presented to the emergency department (ED) with acute onset of severe abdominal pain and distention with associated diffuse tenderness and guarding. Her medical history was significant for a two-year history of fibroids, which contributed to mild menorrhagia. Within 30 minutes of arrival, the patient developed signs of shock with a blood pressure of 89/67 mmHg, heart rate of 115 beats per minute, and a drop in serial hemoglobin measurements from 8.4 g/dL to 6.8 g/dL. Point-of-care ultrasound showed a large amount of free fluid in the abdomen associated with a large abdominal mass originating in the pelvis. Emergent computed tomography (CT) imaging demonstrated a large amount of intra-peritoneal bleeding associated with massive fibroids as shown in Images 1–2. Exploratory laparotomy discovered 3L of hemoperitoneum as well as a roughly 30-week-sized uterus with multiple fibroids, two of which were torsed and actively bleeding. The patient received four units of packed red blood cells, underwent emergent supracervical hysterectomy without additional complications, and was eventually discharged on post-operative day 3. Surgical pathology demonstrated normal endocervical and endometrial tissue, as well as multiple intramural and subserosal leiomyomas measuring up to 17.8 cm in length.

## DISCUSSION

Leiomyomas, often called fibroids, are common in women of reproductive age.^1^–^3^ Hemoperitoneum as a result of spontaneous fibroid rupture or torsion is extremely rare with only one case report found in the emergency medicine literature within the last 20 years and is associated with fibroids greater than 10 cm.^1^–^3^ Other etiologies of spontaneous hemoperitoneum include hepatic and splenic rupture, ovarian cyst and ectopic pregnancy rupture, vascular rupture, and bleeding disorders.^4^ All may have similar initial presentations to the ED with acute onset of abdominal pain and signs of hemorrhagic shock.

## References

[1] Lotterman S (2008). Massive hemoperitoneum resulting from spontaneous rupture of uterine leiomyoma. Am J Emerg Med.

[2] Peng CR, Chen CP, Wang KG (2015). Spontaneous rupture and massive hemoperitoneium from uterine leiomyomas and adenomyosis in a nongravid and unscarred uterus. Taiwan J Obstet Gynecol.

[3] Wong L, Ching TW, Kok TL (2005). Spontaneous hemoperitoneum from a uterine leiomyoma in pregnancy. Acta Obstet Gynecol Scand.

[4] Lucey BC, Varghese JC, Soto JA (2005). Spontaneous hemoperitoneum: causes and significance. Curr Probl Diagn Radiol.

